# Are we capturing individual differences? Evaluating the test–retest reliability of experimental tasks used to measure social cognitive abilities

**DOI:** 10.3758/s13428-025-02606-5

**Published:** 2025-01-31

**Authors:** Charlotte R. Pennington, Kayley Birch-Hurst, Matthew Ploszajski, Kait Clark, Craig Hedge, Daniel J. Shaw

**Affiliations:** 1 School of Psychology, College of Health & Life Sciences, Aston University, Birmingham, B4 7ET UK; 2 School of Social Sciences, University of the West of England, Bristol, UK; 3 Department of Computer Science, College of Science and Engineering, Swansea University, Wales, UK

**Keywords:** Test–retest reliability, Experimental social cognition, Individual differences, Implicit Association Test, Stimulus–Response Compatibility task, Emotional Go/No-Go task, State Affective Empathy task, Dot Perspective-Taking task, Interpersonal Reactivity Index, Explicit bias

## Abstract

Social cognitive skills are crucial for positive interpersonal relationships, health, and wellbeing and encompass both automatic and reflexive processes. To assess this myriad of skills, researchers have developed numerous experimental tasks that measure automatic imitation, emotion recognition, empathy, perspective taking, and intergroup bias and have used these to reveal important individual differences in social cognition. However, the very reason these tasks produce robust experimental effects – low between-participant variability – can make their use as correlational tools problematic. We performed an evaluation of test–retest reliability for common experimental tasks that measure social cognition. One-hundred and fifty participants completed the race-Implicit Association Test (r-IAT), Stimulus–Response Compatibility (SRC) task, Emotional Go/No-Go (eGNG) task, Dot Perspective-Taking (DPT) task, and State Affective Empathy (SAE) task, as well as the Interpersonal Reactivity Index (IRI) and indices of Explicit Bias (EB) across two sessions within 3 weeks. Estimates of test–retest reliability varied considerably between tasks and their indices: the eGNG task had good reliability (ICC = 0.63–0.69); the SAE task had moderate-to-good reliability (ICC = 0.56–0.77); the r-IAT had moderate reliability (ICC = 0.49); the DPT task had poor-to-good reliability (ICC = 0.24–0.60); and the SRC task had poor reliability (ICC = 0.09–0.29). The IRI had good-to-excellent reliability (ICC = 0.76–0.83) and EB had good reliability (ICC = 0.70–0.77). Experimental tasks of social cognition are used routinely to assess individual differences, but their suitability for this is rarely evaluated. Researchers investigating individual differences must assess the test–retest reliability of their measures.

**Supplementary Information:**

The online version contains supplementary material available at 10.3758/s13428-025-02606-5.

## Introduction

Social cognition refers collectively to the broad array of mental operations that we perform when processing and interpreting social information (e.g., others’ verbal and non-verbal expressions; Frith & Frith, 2007, 2012; Happé et al., 2017). The various components of social cognition are thought to comprise both automatic processes involved in social and emotional cue perception and more explicit processes required for using these cues to infer others’ mental and emotional states. Since the efficiency of these socio-cognitive processes determines our behaviour in social interactions, they can have a profound impact on the quality of our interpersonal relationships and, in turn, our physical and mental health (see Holt-Lunstad et al., 2010; Santamaría-García et al., 2020). In this light, accurately capturing individual differences in social cognitive abilities is a crucial endeavour of psychological research; only then can we begin to determine the causes and consequences of their disruption in neurological (Cotter et al., 2018; Henry et al., 2016) and neurodevelopmental disorders (Happé & Frith, 2014; Schilbach, 2016). Achieving this goal requires measurement tools that can reliably capture such individual differences, so the present study evaluated the test–retest reliability of several instruments employed commonly in the field of experimental social cognition.

Numerous experimental tasks have been developed to measure distinct components of social cognition, and a wealth of research has employed these to assess individual differences in socio-cognitive processes (e.g., Bukowski & Samson, 2017; Shaw et al., 2020; see Happé et al., 2017 for an overview). Surprisingly, however, little attention has been paid to whether these tasks are suitable for investigating individual differences. Measures of repeatability – referred to as *test–retest* *reliability* – quantify the stability of a measure over time, and high stability is important for testing between-participant variation. Although estimates of test–retest reliability are evaluated routinely in the development of self-report instruments, they are largely ignored for experimental tasks (Parsons et al., 2019). This is problematic because opposing characteristics make a task suitable for either experimental *or* individual differences research: a phenomenon referred to as the *reliability paradox* (Hedge et al., 2018, 2020). Experimental tasks designed to measure social cognition rely typically on within-participant effects to isolate social-specific processes (e.g., the automatic imitation effect on the Stimulus–Response Compatibility task) from domain-general processes (e.g., overall reaction time). In experimental contexts, the “success” of these tasks is gauged by the extent to which they produce a significant within-subject effect at the group level; when the mean effect is relatively high and between-participant variability is relatively low. However, low between-subject variability causes low reliability for individual differences, thus compromising correlations with other constructs because of the inability to distinguish effectively between individuals on that dimension (Spearman, 1910). Concerns have been raised about the reliability of experimental tasks used commonly in several fields, including cognitive control (Hedge et al., 2018), visual cognition (Clark et al., 2022) and functional imaging (Infantolino et al., 2018). Quantifying the reliability of experimental measures is a common requirement for the optimal design and interpretation of individual differences studies. In the field of social cognition, however, the test–retest reliability of many experimental tasks is currently unknown. Researchers often assume that components of social cognition reflect (at least in part) psychological processes or attitudes that are stable over time (see Happé et al., 2017; Nosek et al., 2011; Shaw et al., 2020; Schimmack, 2021a, 2021b). There is also considerable interest in using measures of social cognitive abilities as tools for prediction and early detection of future psychopathology (e.g., Gur & Gur, 2015), which entails that they do not solely reflect situational or temporary states. We focus on test–retest reliability for these very reasons. In the sections that follow, we describe the constituent components of social cognition that have been the focus of many individual difference studies in this literature – namely automatic imitation, perspective taking, empathy, emotion recognition, and implicit intergroup attitudes – and outline the experimental tasks designed specifically, and employed commonly, to measure them.

One core component of social cognition that is investigated frequently is that of automatic imitation – humans have an involuntary tendency to mimic the behaviours of one another, which is believed to reflect the automatic activation of overlapping self- and other-action representations in the motor system (Cracco et al., 2018a; Heyes, 2011). Such behavioural imitation has been shown to increase feelings of affiliation and cooperation among individuals, thereby serving an important function during social interactions (Chartrand & Lakin, 2013). Studies of automatic imitation commonly employ an adapted version of the experimental Stimulus–Response Compatibility (SRC) procedure, wherein the topographic features of observed actions either facilitate similar or interfere with dissimilar responses (Brass et al., 2001). In a seminal study employing this task, Brass et al. (2001) instructed participants to lift their index or middle fingers in response to a number cue whilst watching either topographically similar (congruent) or dissimilar (incongruent) finger movements performed by a stimulus hand. Findings indicated that participants were faster and more accurate in executing finger movements directed by the task-relevant cue when simultaneously observing task-irrelevant congruent compared with incongruent stimulus movements. Meta-analyses indicate that this automatic imitation effect is strong and robust (Cracco et al., 2018a), supporting the proposition that movement observation exerts an influence on movement execution by automatically engaging corresponding action representations (Brass & Heyes, 2005). Measures of individual differences in automatic imitation have since been used to predict the severity of various disorders and personality styles characterised partly by dysfunctional interpersonal behaviour, including autism (e.g., Spengler et al., 2010) and narcissism (Obhi et al., 2014).

Another element of social cognition important for interpersonal behaviour is visual perspective taking – the process through which we can infer what is and is not visible to someone else when their viewpoint differs from our own. This ‘Level-1 perspective taking’ (Flavell et al., 1978; Santiesteban et al., 2012a, 2012b; Spengler et al., 2010,1) requires us to detach ourselves from our own visual representation of the world to infer what another person can or cannot see (Bukowski, 2018; Bukowski et al., 2015; Epley et al., 2004). Experimental tasks such as the Dot Perspective-Taking (DPT) task have been developed to measure this automatic capacity (Samson et al., 2010), wherein participants are required to judge the number of items visible from their own or another person’s viewpoint when the two perspectives are identical or different. Performance on this task shows that perspective taking is susceptible to both egocentric and altercentric misattributions; in their seminal study, Samson et al. (2010) found that whilst participants were quicker to make self- relative to other-perspective judgments, for the former they could not easily ignore the perspective of somebody else. Individual differences in this capacity have been found in relation to alexithymia, schizotypy, multilingualism, willingness to forgive, and proneness to guilt (Langdon & Coltheart, 2001; Leith & Baumeister, 2008; Ryskin et al., 2015). Moreover, using an individual differences approach, Bukowski and Samson (2017) distinguished between individuals with ‘good’ and ‘poor’ perspective-taking ability based on their propensity to efficiently handle conflicting viewpoints or focus on their own or the other person’s perspective.

Successful social interactions also require us to infer, share and behave compassionately towards the emotional states of others, a sociocognitive process referred to as empathy*.* Research in this area distinguishes between cognitive and affective empathy – the former referring to the ability to understand another person’s emotional state, and the latter referring to our capacity to experience or share in another’s emotional state vicariously (Dziobek et al., 2008). The Multifaceted Empathy test (Dziobek et al., 2008) and the State Affective Empathy task (Brown et al., 2006) were designed to dissociate these two dimensions, and performance on these tasks indicates reduced cognitive empathy in individuals with certain neurodevelopmental disorders (e.g., autism and Aspergers; Quinde-Zlibut et al., 2021). Davis (1980, 1983) also proposed that empathy was a multifaceted rather than unitary construct and developed the frequently used Interpersonal Reactivity Index (IRI) – a self-report instrument that dissociates empathic concern, personal distress, perspective taking, and fantasy. Supporting our point regarding the validation of self-reports over experimental tasks, the IRI has undergone substantial validation (e.g., Carey et al., 1988; Raimondi et al., 2023) and has been shown to have excellent test–retest reliability (Davis, 1983), making it suitable for the investigation of individual differences. However, to our knowledge, no such assessment of the Multifaceted Empathy test or State Affective Empathy task has been performed.

Our ability to empathise relies on us being able to accurately identify the emotions expressed by others (see Coll et al., 2017) – a related socio-cognitive process referred to as emotion recognition (Besel & Yuille, 2010). The frequently used Emotional Go/No-Go task (Tottenham et al., 2011) distinguishes between emotion recognition (the ability to discriminate between different emotions in oneself and others) and emotion regulation (the ability to maintain cognitive control in the context of interfering emotional information). This distinction is important because recognising the emotional states of others modifies our behaviour towards them, yet such emotion-driven behaviours can be ill-suited to certain social contexts and must therefore be regulated. Evidence from developmental research suggests that emotion recognition skills develop within the first year of life (Nelson & Dolgin, 1985) and continue to develop throughout childhood and adolescence at which point they become relatively stable traits (Thomas et al., 2007; Tottenham et al., 2011). In this light, individual differences in early emotion recognition ability are thought to impact more sophisticated socio-cognitive processes necessary for emotional and social understanding. Importantly, then, impairments in this ability are evident in certain neurodevelopmental disorders (Jones et al., 2011).

A large body of research also suggests that our interpersonal behaviour can be influenced by the attitudes and biases we hold towards different social groups (see Happé et al., 2017; Nosek et al., 2011). The Implicit Association Test (IAT; Greenwald et al., 1998) is the most frequently used task proposed to measure individual differences in intergroup attitudes, and much work has focused on correlates with (inter)group behaviour and discrimination (see Greenwald et al., 2009; Oswald et al., 2013 for meta-analyses). Indeed, some research in the field of social cognition has shown that individual differences in implicit racial attitudes, as measured by the race-based IAT, appear to be related to imitative tendencies, perspective taking, empathy, and emotion recognition (e.g., Azevedo et al., 2012; Fabi & Leuthold, 2018; Rauchbauer et al., 2016; Schneider et al., 2018; Wang et al., 2014). Recently, however, the validity of the IAT as an individual difference measure has come under intense scrutiny (see Pennington et al., 2023; Schimmack, 2021a, 2021b for overviews), with researchers noting substantial noise around the point estimates of IAT scores (Connor & Evers, 2020; Cummins & Hussey, 2023; Klein, 2020).

Despite the wealth of research that has employed the aforementioned experimental tasks to measure individual differences in social cognition, we found no studies that report the test–retest reliability of the Stimulus–Response Compatibility task, Dot Perspective-Taking task, Multifaceted Empathy task, State Affective Empathy task, and Emotional Go/No-Go task.^2^ An exception to this is the Implicit Association Test, for which test–retest reliability has been estimated in numerous studies but is generally agreed to be lower than desired for an individual difference measure (Gawronski et al., 2017; Lai & Wilson, 2021; Lane et al., 2007). Therefore, the current study provides the first investigation into the test–retest reliability of a large battery of experimental social cognition tasks. Since self-report measures have been found to typically yield higher test–retest reliability than experimental ones (see Hedge et al., 2018; Zeynep Enkavi et al., 2019), we also set out to compare the estimates derived from our experimental tasks with two self-report measures – the Interpersonal Reactivity Index and a frequently used measure of explicit intergroup bias.

## Method

### Transparency and data availability statement

All experimental materials, raw data, and analysis scripts are publicly available on the Open Science Framework: https://osf.io/q569f/. In the following sections, we report all measures, manipulations, and exclusions. The data reported herein for session 1 represent a sub-sample of participants from Pennington et al., (2023; Experiment 1) who were recruited from a single university in the United Kingdom and successfully re-recruited for a second session to assess the test–retest reliability of experimental social cognition measures.

### Participants

We recruited 162 participants to take part in a two-session lab-based study investigating relationships between different measures of social cognition, the two sessions taking place 2–3 weeks apart. Twelve participants did not return for the second session (attrition rate = 7.41%), resulting in a final sample of 150 participants (*M*age = 20.75, *SD* = 0.33, 127 females, 124 White) who were reimbursed with course credits. All participants met the inclusion criteria of reporting normal or corrected-to-normal vision and no neurological or psychiatric disorders. The experimental protocol was given ethical approval from the Institutional Review Board at the University of the West of England (REF: HAS.18.07.21). All participants provided written informed consent.

The sample size was determined solely by the largest number of participants we could recruit based on time constraints. To evaluate the adequacy of this sample size, we conducted a simulation to determine the average width of the 95% confidence interval for different assumptions about the true level of reliability (in line with Clark et al., 2022; Doros & Lew, 2010). First, we simulated two correlated variables for a population of 100,000 individuals. Second, we took a random sample of 102 individuals and 148 (our lowest and highest samples based on complete task data across sessions; see “Results” for all data exclusions) and calculated the intraclass correlation (ICC), along with the 95% confidence intervals. We then repeated this second step 10,000 times and took an average of these values. The results of these simulations are provided in Table 1.

**Table 1:** Simulated average ICC and 95% confidence intervals

True R	*N* = 102	*N* = 148
0.4	0.40 [0.22 0.55]	0.40 [0.25 0.52]
0.6	0.60 [0.45, 0.71]	0.60 [0.48, 0.69]
0.8	0.80 [0.71, 0.86]	0.80 [0.73, 0.85]

Each entry is based on 10,000 random samples from a population of 100,000. *N* = 102 is the lowest sample size with complete data across sessions and *N* = 148 is the largest sample size

We adopted this approach because a traditional power analysis assumes the goal is to reject the null hypothesis (ICC = 0). When estimating reliability, however, the accuracy of the estimate is more important – knowing that the reliability of a task is ICC = 0.8 rather than ICC = 0.5 could influence our decisions about whether to use it. The 95% confidence interval contains the values that are consistent with our data, so these should be narrow enough to exclude values that would change our conclusions substantially. The average widths reported in Table 1 are less than or equal to 0.33 (0.55–0.22) and do not span more than two of the traditional reliability thresholds (see Analytic strategy). In other words, if a measure has moderate reliability, then we can reject the conclusion that it might have excellent reliability and vice versa. Based on these simulations, we conclude that our sample size is sufficient.

## Measures and procedure

**Fig. 1:**
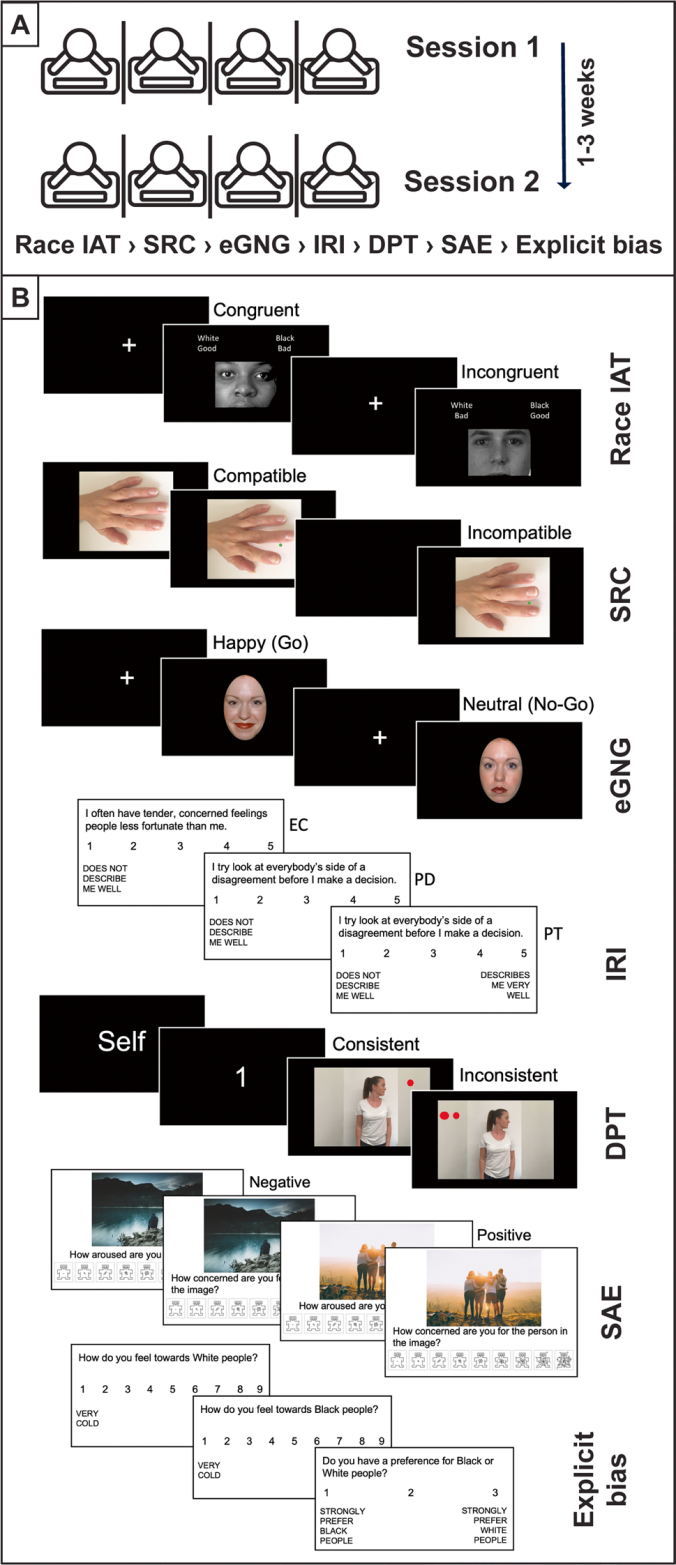
Test–retest experimental procedure and tasks. Note. Race IAT = Race Implicit Association Test; SRC = Stimulus–Response Compatibility task (left hand stimulus rotated counterclockwise); eGNG = Emotional Go/No-Go task; IRI = Interpersonal Reactivity Index, EC = Empathic Concern, PD = Personal Distress, PT = Perspective Taking; DPT = Dot Perspective-Taking task; SAE = State Affective Empathy task; Explicit bias = explicit intergroup bias

In both sessions, participants sat 57 cm from a standard computer monitor and completed a computerised battery of experimental tasks employed frequently to measure specific socio-cognitive processes: the Race Implicit Association Test, Stimulus–Response Compatibility task, Emotional Go/No-Go task, State Affective Empathy task, and Dot Perspective-Taking task. As these tasks are commonly administered alongside self-report measures of social cognition, participants also completed the Interpersonal Reactivity Index and a measure of explicit intergroup bias. All experimental tasks and self-report measures were programmed in MATLAB (R2017b; MathWorks Inc, Natick, MA, USA) using the Cogent toolbox (v1.31) and were presented in the fixed order they appear below as recommended when assessing test–retest reliability (Clark et al., 2022; Goodhew & Edwards, 2019; Hedge et al., 2018). Up to four participants were tested at a multi-testing station separated by dividers. In each session, the battery took approximately 1 h to complete, and participants were instructed to take breaks between tasks to reduce fatigue (Fig. 1A).

### Experimental tasks

#### Race Implicit Association Test (Race IAT) 

The standard Race IAT (Greenwald et al., 1998) was employed to measure implicit intergroup bias. In this task, participants are required to classify stimuli depicting White and Black faces and/or positive and negative words into superordinate categories (White, Black, Good, Bad) as fast as possible. There are seven blocks comprising five practice blocks (20 trials each) and two critical test blocks (40 trials each). In one of these critical blocks, participants are instructed to categorise serially presented White faces and positive words into the category “White/Good” and Black faces and negative words into the category “Black/Bad” using two response keys. In the other counterbalanced critical block, the response mapping is switched, and participants categorise White faces and negative words into “White/Bad” and Black faces and positive words into “Black/Good” (see Fig. 1B). The task is based upon the theoretical premise that people should be quicker to categorise concepts that are closely associated in memory, thus revealing individual differences in implicit racial bias (Greenwald et al., 1998).

Stimuli were selected from the Project Implicit website and comprised six grey-scale photographs of White and six Black people, with an equal number of females and males. Eight positive words (attractive, glad, delightful, spectacular, excitement, celebrate, fantastic, triumph) were matched with eight negative words (yucky, disaster, awful, negative, selfish, dirty, scorn, hurtful). Within all blocks, the inter-trial interval was 1000 ms, consisting of a white fixation cross. Incorrect responses were signalled by a red cross, displayed until a correct response was given. The main dependent variable of interest is the IAT *d*-score, which was calculated according to the conventional revised scoring algorithm (Greenwald et al., 2003, pp. 214). Here, positive scores correspond to a pro-White/anti-Black bias and negative scores correspond to a pro-Black/anti-White bias.

#### Stimulus–Response Compatibility (SRC) task 

The SRC task (Brass et al., 2001) was employed to measure automatic imitation. Participants are required to execute finger-lifting actions with their right-hand in response to an imperative stimulus (coloured dot) whilst observing task-irrelevant index- or middle-finger movements performed simultaneously by a left-hand stimulus. To isolate imitative from simple spatial compatibility effects, researchers have started to rotate this left-hand stimulus 90° counterclockwise, thus placing observed and executed finger movements orthogonal to one another (e.g., Cook & Bird, 2011). However, recent research indicates that this stimulus is then confounded by orthogonal compatibility and instead recommends the use of a *right*-hand stimulus rotated 90° counterclockwise (Czekóová et al., 2021; Shaw et al., 2017). For this reason, we employed two versions of this task: one with a left-hand and one with a right-hand stimulus rotated 90° counterclockwise, referred to herein as “SRC left-hand” and “SRC right-hand”, respectively.

During this task, participants saw a stimulus hand resting flat on a surface from a bird’s-eye view and were instructed to depress both the left and right directional arrows on the computer keyboard using their index and middle finger of their right-hand, respectively. After a randomised interval of either 800, 1600, or 2400 ms, the stimulus hand moved to an endpoint of either an index- or middle-finger extension and a green or red dot was presented. The coloured dot served as an imperative stimulus that signalled whether the participant should lift their own index (green dot) or middle finger (red dot), thereby releasing the corresponding key. A blank screen was presented for 1000 ms between trials. Participants completed two blocks of 72 trials, one utilising the SRC left-hand and the other utilising the SRC right-hand. Each block comprised 24 compatible trials (i.e., the imperative stimulus signalled a movement that corresponded to the stimulus hand), 24 incompatible trials (i.e., the signalled movement was opposite) and 24 baseline trials (i.e., a movement was signalled, but not observed). Both RT and accuracy were recorded. The main dependent variable of interest in this paradigm is the automatic imitation effect, which was derived by subtracting mean RT on compatible from incompatible trials. Higher values correspond to greater automatic imitation.

#### Emotional Go/No-Go (eGNG) task 

The eGNG task (Tottenham et al., 2011) was employed as a measure of emotion recognition (i.e. accurately discriminating between different emotions) and emotion regulation (i.e., inhibiting a prepotent behavioural response in the context of emotional information). Participants are presented with a sequence of faces in rapid succession and are required to respond as quickly as possible, by pressing the space bar, when a pre-specified facial expression appears. The eGNG task comprised six blocks of 40 trials. In three emotional “Go” blocks, participants were required to respond to faces depicting happy, sad, and angry emotions, and inhibit their responses to neutral faces (“No-Go” trials). During three non-emotional “Go” blocks, these instructions were reversed. Face stimuli were selected from the NimStim database (Tottenham et al., 2009) and were cropped to remove any hair. In each block, a specific emotional expression was paired with neutral faces (angry-neutral, neutral-angry, happy-neutral, neutral-happy, sad-neutral, neutral-sad) with blocks presented in a pseudorandomised order. Each trial began with a fixation-cross presented for 1000–2000 ms, followed by a face stimulus presented for 500 ms. Go trials occurred frequently (70%) to evoke a prepotent tendency to respond, and the order of Go and No-go trials were pseudorandomised to ensure that no two No-go trials occurred successively within a block. In line with Tottenham et al. (2011), we extracted two dependent variables: as a measure of emotion recognition, we calculated *d-*prime by subtracting the *z*-transformed false alarm rate from the *z*-transformed hit rate across all trials, which provides an index of accuracy accounting for response bias. As a measure of emotion regulation, we calculated the false alarm rate averaged across emotional No-Go trials. Higher scores correspond to better emotion recognition and poorer emotion regulation, respectively.

#### Interpersonal Reactivity Index (IRI)

 We employed the IRI (Davis, 1983) as a self-report measure of trait empathy and its composite dimensions. Specifically, this 28-item comprises three relevant sub-scales of interest: Perspective Taking (PT; the tendency to adopt the psychological perspective of others), Empathic Concern (EC; adopting “other-oriented” feelings of sympathy and concern in response to the suffering of others), and Personal Distress (PD; self-orientated feelings of personal anxiety and unease in tense interpersonal settings). For completeness and data reuse, in the tables below we also report data for the subscale of Fantasy (FS; the tendency to transpose oneself imaginatively into the feelings of fictitious characters) but do not discuss this further because it was not considered to correspond with our other measures of social cognition. Participants responded to statements (e.g., “I often have tender, concerned feelings for people less fortunate than me” [EC]) on a five-point scale (1 = Does not describe me well, 5 = Describes me very well). Each sub-scale had acceptable internal reliability in the current study (session 1, McDonald’s *ω*, PT = 0.80, EC, = 0.78, PD = 0.71) and a total score was computed for each. Higher scores correspond to higher self-reported PT, EC, and PD, respectively.

#### Dot Perspective-Taking (DPT) task

The DPT task (Samson et al., 2010) was employed as a measure of Level 1 perspective taking. In line with Langton (2018), our experimental stimuli depicted real human actors and realistic settings rather than computer-generated avatars and settings.^3^ During this task, participants see a picture of a room with a matched-sex actor facing either the left or right wall. On each trial, a number of red discs (0–3) are displayed on one or both walls and participants are asked to judge how many discs can be seen from either their own (“Self”) or the actor’s perspective (“Other”). During “Consistent” trials (CON), both the participant and actor can see the same number of discs, and during “Inconsistent” trials (INCON), the number of discs differs between the participant and actor. Participants are required to indicate whether the digit specified matches or mismatches the number of dots visible from the given perspective.

In each of four blocks, there were 48 experimental and four filler trials, the former divided equally among the factorial combination of Perspective (Self vs. Other), Consistency (Consistent vs. Inconsistent), and Trial Type (Matching vs. Mismatching). Each trial began with a fixation cross presented for 750 ms. After 500 ms, the word ‘Self’ or ‘Other’ was presented for 750 ms, instructing the participant to take their own perspective or that of the actor’s. After another 500 ms, a digit between one and three was presented in the middle of the screen for 750 ms. During filler trials, no discs were displayed on the wall thereby always requiring a “mismatch” response. Participants were required to respond within 2000 ms by pressing one of two assigned keys. The order of trials was pseudorandomised and blocks were counterbalanced across participants. Only “match” responses are entered into the analyses as per Samson et al. (2010).

In line with Bukowski and Samson (2017), we computed three dependent variables that are proposed to capture individual differences in perspective taking. Importantly, however, because the error rate in our data exceeded 10%, we could not calculate inverse efficiency scores; instead, we used RTs (ms), which aligns with most of the research using this task (e.g., Samson et al., 2010). The first dependent variable is the single-dimension index (SDI), which measures participant’s ability to inhibit their own perspective to correctly consider the other person’s differing perspective. This was calculated by averaging RTs across “Other-Inconsistent” trials, with higher values representing greater difficulty in taking another person’s differing perspective (“egocentric interference”). The second variable is the conflict index, which measures interference between self and other perspectives. This was calculated by subtracting the average RT on “Consistent” from “Inconsistent” trials, with higher scores representing greater difficultly in handling conflicting perspectives. The third variable is the focus index, which measures the relative ease of judging the self or other perspective. This was calculated by subtracting average RT on “Other” perspective trials from “Self” perspective trials, with positive values representing better performance in taking the other’s perspective (“altercentric interference”).

#### State Affective Empathy (SAE) task 

The SAE task was designed based on the tasks described by Brown et al. (2006) and Dziobek et al. (2008) to measure state empathic concern and arousal. Participants viewed 38 images depicting White people expressing negative (*n* = 13^4^), positive (*n* = 14) and neutral (*n* = 10) emotions in various contexts. Photographs were selected from the International Affective Picture System (IAPS; Lang et al., 2008) and, importantly, normative ratings of valence and arousal significantly differed between the positive and negative images (*M*_POS-VALENCE_ = 6.96, *SD* = 0.60; *M*_NEG-VALENCE_ = 2.50, *SD* = 0.75, *p* < 0.001, *M*_POS-AROUSAL_ = 4.72, *SD* = 0.94; *M*_NEG-AROUSAL_ = 5.54, *SD* = 1.24, *p* = 0.03). After viewing each image, participants were asked two questions specifically designed to measure affective empathy (Dziobek et al., 2008): “How concerned are you for the person in the image?” and “How aroused are you when viewing this image?”. Responses were recorded on a nine-point Self-Assessment Manikin (1 = Not very much, 9 = Very much). We focus our analyses on *empathic concern* and *arousal* elicited by the positive and negative images only; the former is computed by summing responses to the first question, and the latter is computed by summing responses to the second question. We refer to these variables herein as Concern_*POS*_, Concern_*NEG*_, Arousal_*POS*_ and Arousal_*NEG*_. Higher values correspond to greater affective empathy on each of these subscales.

#### Explicit Intergroup Bias 

Explicit intergroup (racial) bias was measured using three self-report questions from Greenwald et al. (2009). Participants first responded to two questions “*How do you feel towards White people?*” and “*How do you feel towards Black people?*” on a ten-point scale (0 = Very cold, 9 = Very warm). They then responded to a third question: “*Do you have a preference for Black or White people?*” on a three-point scale (1 = Strongly prefer Black people, 3 = Strongly prefer White people). Subtracting responses to the second question from the first provide an index of relative warmth towards White people, referred to herein as “Warmth_*W*_”. Responses to the third question provide a separate measure of relative preference towards White people, referred to herein as Preference_*W*_. Higher values on Warmth_*W*_ and values greater than 2 on Preference_*W*_ correspond to a Pro-White/anti-Black bias.

## Results

### Analytic strategy

Individual task or questionnaire data were excluded from analyses in two iterative steps: first, if either timepoint was missing, and second, if participants scored below 50% accuracy on the SRC or DPT tasks (chance performance). File S1 details all data exclusions. After these, the final sample size for each constituent task was as follows: Race IAT, *n* = 147; SRC task left-hand stimulus, *n* = 141; SRC task right-hand stimulus, *n* = 102; eGNG task, *n* = 148; IRI, *n* = 144; DPT task, *n* = 129; SAE task, *n* = 145; and explicit bias, *n* = 144.

To provide a general overview of how participants performed in each task, first we report descriptive statistics for session 1 and 2 and several performance checks. For the Race IAT we used a one-sample *t* test against a baseline of zero, and for the SRC, DPT, and SAE tasks we used paired-samples *t* tests (two-tailed). We then report the test–retest reliabilities for each task and its respective measurement indices. Reliabilities take the form of the ICC, using a two-way mixed-effects model for absolute agreement that can account for various sources of variance separately (Koo & Li, 2016). All ICC estimates were computed using MATLAB version 9.14 (MathWorks Inc, 2023). The ICC takes the form:

$$\text{ICC}= \frac{\text{Variance between participants}}{\text{Variance between participants}+\text{Error variance}+\text{Variance between sessions}}$$

ICC estimates range from 0 to 1 and typical interpretations are as follows: Excellent =  > 0.80, good = 0.60–0.80, moderate = 0.40–0.60, and poor =  < 0.40 levels of reliability (Cicchetti & Sparrow, 1981; Fleiss, 1981; Landis & Koch, 1977). Additionally, we report the standard error of measurement (SEM) for each measure, which is analogous to the standard error of the mean. As an alternative approach to estimating test–retest reliability, we also report the Spearman’s rho correlation for each key measure. Spearman’s rho and 95% confidence intervals were calculated using JASP (JASP Team, 2023; Version 0.16.3).

### Task performance

The descriptive statistics for each of the task indices for sessions 1 and 2 are reported in bold within Table 2. In addition, we also report secondary indices for the Race IAT, SRC, eGNG, and DPT tasks that are also used in the literature.

**Table 2:** Means and standard deviations for indices of each social cognition task with main dependent variables in bold

Task	Index	Session 1		Session 2	
		*M*	*SD*	*M*	*SD*
Race IAT	Accuracy	0.94	0.04	0.93	0.05
	***d*-score**	0.37	0.35	0.36	0.34
SRC right-hand	Accuracy	0.89	0.08	0.92	0.06
	Compatible RT (ms)	549.06	80.48	532.35	65.43
	Incompatible RT (ms)	559.30	78.41	531.60	70.92
	**SRC effect (ms)**	10.23	40.43	−0.76	41.39
SRC left-hand	Accuracy	0.90	0.07	0.91	0.07
	Compatible RT (ms)	563.69	71.24	552.09	68.03
	Incompatible RT (ms)	591.10	79.28	578.57	68.27
	**SRC effect (ms)**	27.41	43.05	26.48	39.00
eGNG	***d*-prime**^a^	0.00	1.50	0.00	1.55
	**FA rate**	0.31	0.14	0.32	0.16
	Hit rate	0.90	0.06	0.90	0.06
IRI	**EC score**	20.97	4.85	20.49	4.78
	**PD score**	13.69	4.70	12.96	5.15
	**PT score**	18.17	4.86	18.35	4.53
	FS score	17.30	5.80	17.20	5.70
DPT	Accuracy	0.74	0.08	0.77	0.07
	CON RT (ms)	753.45	145.23	673.48	138.43
	INCON RT (ms)	837.94	158.71	755.91	152.86
	SELF RT (ms)	780.24	145.49	711.04	143.10
	OTHER RT (ms)	811.15	155.17	718.35	145.89
	**SDI (ms)**	864.87	177.27	766.67	163.09
	**Focus index (ms)**	– 60.00	140.71	– 14.61	118.01
	**Conflict index (ms)**	164.20	174.79	164.87	141.66
SAE	**Concern**_***POS***_** score**	1.65	0.84	1.71	0.91
	**Concern**_***NEG***_** score**	5.72	1.40	5.45	1.60
	**Arousal**_***POS***_** score**	2.78	1.41	2.52	1.48
	**Arousal**_***NEG***_** score**	3.62	1.85	3.36	1.80
Explicit bias	**Warmth**_***W***_	0.04	1.40	−0.06	1.49
	**Preference**_***W***_	2.04	0.31	2.04	0.29

Race IAT = Race Implicit Association Test; SRC right-hand = Stimulus–Response Compatibility task with right-hand stimulus rotated counterclockwise; SRC left-hand = Stimulus–Response Compatibility task with left hand stimulus rotated counterclockwise; eGNG = Emotional Go/No-Go task; IRI = Interpersonal Reactivity Index, EC = Empathic Concern, PD = Personal Distress, PT = Perspective Taking, FS = Fantasy; DPT = Dot Perspective-Taking task, SDI = Single-dimension index; SAE = State Affective Empathy task; Explicit bias = Explicit intergroup bias. Primary measures are highlighted in bold.

^a^Mean standardised value (*z*-transformed hit rate minus *z*-transformed false-alarm rate).

For the four experimental tasks, all expected effects were observed and replicated those reported in the original studies. Specifically, participants exhibited a pro-White/anti-Black bias (*M* = 0.36, *SD* = 0.30) on the Race IAT, with the average *d*-score aggregated across sessions differing significantly from zero (*t*(146) = 14.71, *p* < 0.001, *d*_*z*_ = 1.21). On the SRC task, participants were quicker to respond to Compatible (*M* = 557.89, *SD* = 62.88) relative to Incompatible trials for the (orthogonally confounded) left-hand stimulus (*M* = 584.84, *SD* = 66.65; *t*(140) = 9.71, *p* < 0.001, *d*_*z*_ = 0.82), but the difference was non-significant between Incompatible (*M* = 545.45, *SD* = 65.76) and Compatible trials for the (non-confounded) right-hand stimulus (*M* = 540.71, *SD* = 66.46; *t*(101) = 1.58, *p* = 0.117, *d*_*z*_ = 0.16). This latter result is expected given the absence of orthogonal confounds that has been shown to reduce – or even partially reverse – the automatic imitation effect (Czekóová et al., 2021). On the eGNG task, participants had significantly higher false alarm rates on emotional (*M* = 0.32, *SD* = 0.14) relative to neutral No-Go trials (*M* = 0.14, *SD* = 0.10), *t*(149) = 21.55, *p* < 0.001, *d*_*z*_ = 1.76). On the DPT task, participants responded quicker to Consistent^5^ (*M* = 713.46, *SD* = 130.87) relative to Inconsistent trials (*M* = 796.93, *SD* = 144.00; *t*(128) = 15.30, *p* < 0.001, *d*_*z*_ = 1.35), and to Self (*M* = 745.64, *SD* = 132.98) compared to Other trials (*M* = 764.75, *SD* = 139.91; *t*(128) = 4.23, *p* < 0.001, *d*_*z*_ = 0.37). On the SAE task, self-reported affective empathy was greater for negative (*M* = 4.54, *SD* = 1.28) relative to positive valence images (*M* = 2.17, *SD* = 0.91; *t*(144) = 21.61, *p* < 0.001, *d*_*z*_ = 1.79). File S1 provides full details.

### Task reliabilities

Table 3 summarises the ICCs for the primary task indices. We also report Spearman’s rho to ensure that, if our interpretations are driven by outliers or influential data points, these are generally similar to the ICCs.

**Table 3:** Intraclass correlation coefficient (ICC) and Spearman’s rho for indices of each social cognition task

Task	Index	ICC [95% CI]	Rho [95% CI]
Race IAT	*d*-score	0.49 [0.36, 0.60]	0.51** [0.38, 0.62]
SRC right-hand	SRC effect (ms)	0.09 [– 0.10, 0.28]	0.05 [– 0.14, 0.25]
SRC left-hand	SRC effect (ms)	0.29 [0.13, 0.43]	0.26* [0.10, 0.41]
eGNG	*d*-prime	0.63 [0.53, 0.72]	0.62** [0.51, 0.71]
	FA rate	0.69 [0.60, 0.77]	0.69** [0.59, 0.76]
IRI	EC score	0.81 [0.75, 0.86]	0.73** [0.65, 0.80]
	PD score	0.83 [0.77, 0.88]	0.83** [0.77, 0.87]
	PT score	0.76 [0.68, 0.82]	0.73** [0.64, 0.80]
	FS score	0.83 [0.77, 0.88]	0.83** [0.76, 0.88]
DPT	SDI (ms)	0.60 [0.21, 0.78]	0.69** [0.58, 0.77]
	Focus index (ms)	0.24 [0.08, 0.39]	0.24* [0.07, 0.39]
	Conflict index (ms)	0.28 [0.12, 0.44]	0.30** [0.14, 0.45]
SAE	Concern_*POS*_ score	0.56 [0.44, 0.66]	0.68** [0.58, 0.76]
	Concern_*NEG*_ score	0.69 [0.58, 0.76]	0.69** [0.59, 0.77]
	Arousal_*POS*_ score	0.67 [0.56, 0.75]	0.67** [0.57, 0.75]
	Arousal_*NEG*_ score	0.77 [0.69, 0.83]	0.76** [0.68, 0.82]
Explicit bias	Warmth_*W*_	0.70 [0.61, 0.78]	0.71** [0.62, 0.78]
	Preference_*W*_	0.77 [0.69, 0.76]	0.77** [0.69, 0.83]

* *p* < 0.01, ** *p* < 0.001. Race IAT = Race Implicit Association Test; SRC right-hand = Stimulus–Response Compatibility task with righthand stimulus; SRC left hand = Stimulus–Response Compatibility task with left-hand stimulus; eGNG = Emotional Go/No-Go task; IRI = Interpersonal Reactivity Index, EC = Empathic Concern, PD = Personal Distress, PT = Perspective Taking, FS = Fantasy; DPT = Dot Perspective-Taking task; SDI = Single-dimension index; SAE = State Affective Empathy task; Explicit bias = explicit intergroup bias

**Fig. 2:**
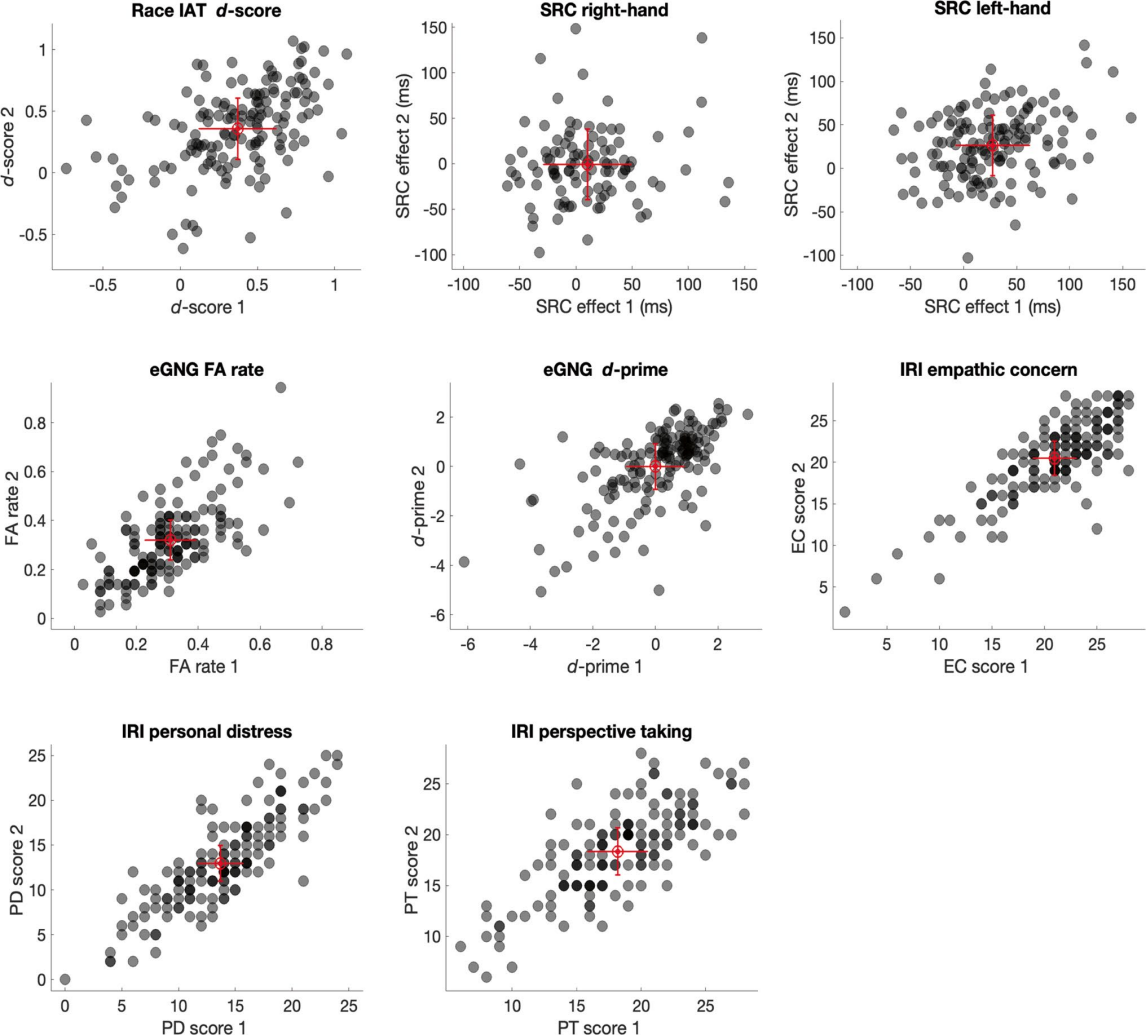
Scatterplots for indices from the Race IAT, SRC, and eGNG tasks, and the IRI. *Note.* Race IAT = Race Implicit Association Test (*n* = 147); SRC right hand = Stimulus–Response Compatibility task with right-hand stimulus rotated counterclockwise (*n* = 102); SRC left hand = Stimulus–Response Compatibility task with left-hand stimulus rotated counterclockwise (*n* = 141); eGNG = Emotional Go/No-Go task (*n* = 148); IRI = Interpersonal Reactivity Index (*n* = 144), EC = Empathic Concern, PD = Personal Distress, PT = Perspective Taking. *Red markers* indicate mean group performance from sessions 1 and 2. *Error bars* show ± standard error of measurement (SEM). *Black markers* indicate individual participant scores

The self-report indices of trait empathy using the IRI demonstrated some of the highest levels of reliability (Table 3): the PT subscale exceeded a standard of good reliability (0.60), and the EC and PD subscales exceeded a level of excellent reliability (> 0.80). For the eGNG task, the indices of emotion recognition (*d*-prime) and emotion regulation (FA rate) both showed good levels of reliability. The Race IAT *d*-score exceeded a moderate level of reliability (> 0.40). The SRC task showed the lowest levels of reliability: responses to both the left (orthogonally confounded) and right-hand stimulus (non-confounded) had poor levels of reliability (< 0.40). Owing to the wide range in participant accuracy on the SRC task, we also report the test–retest reliabilities for participants who performed above 70% and 80% accuracy in File S1, which do not considerably change these estimates. Scatterplots are shown in Fig. 2. As a comparison of the error variance relative to the between-participant variance, each scatterplot shows the standard error of measurement (SEM). A large SEM relative to the between-subject variance contributes to poor reliability.

**Fig. 3:**
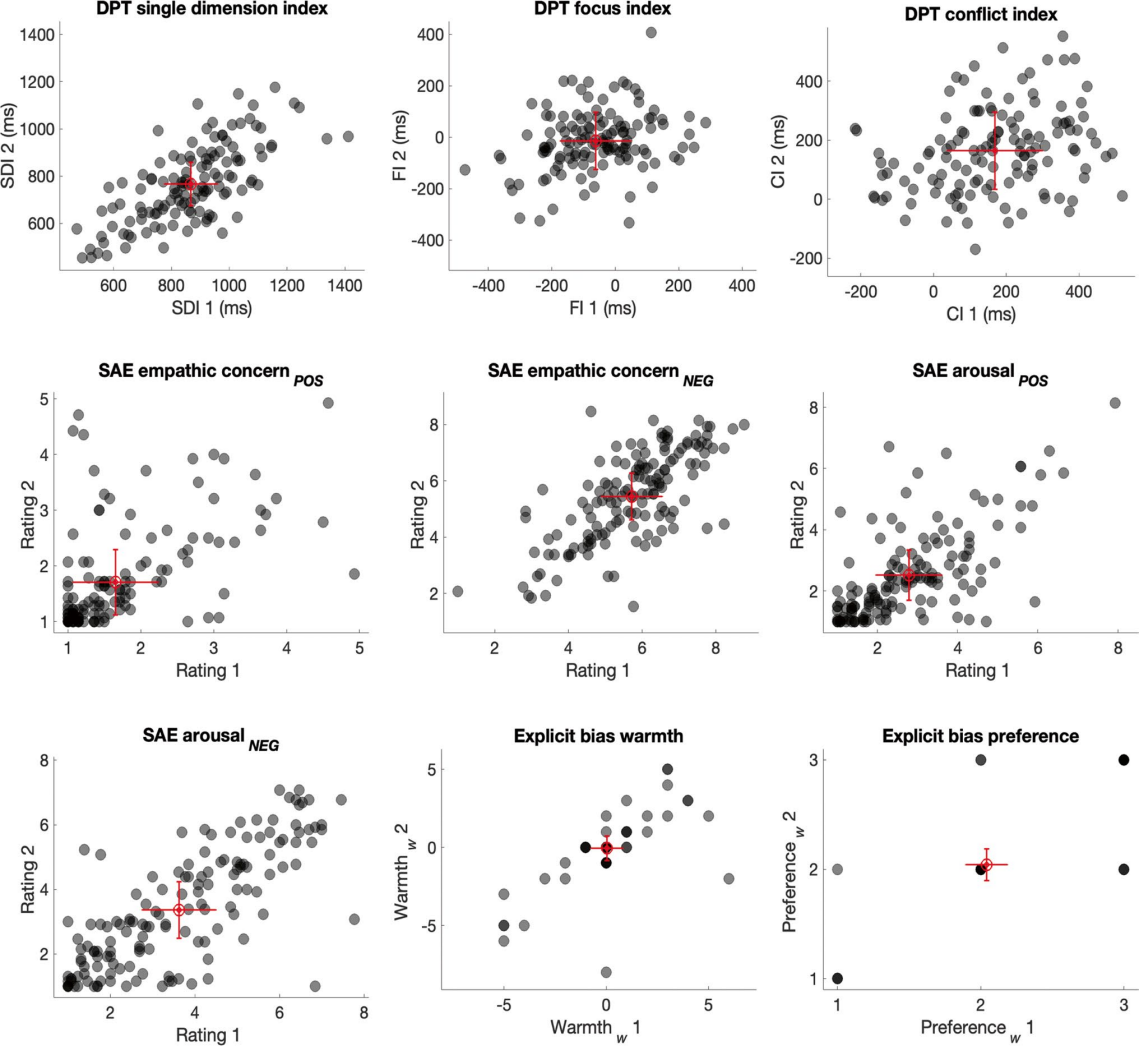
Scatterplots for indices from the DPT task, SAE task and Explicit bias. *Note.* DPT = Dot Perspective-Taking task (*n* = 129); SAE = State Affective Empathy task (*n* = 145); Explicit bias = Explicit intergroup bias (*n* = 144). *Red markers* indicate mean group performance from sessions 1 and 2. *Error bars* show ± standard error of measurement (SEM). *Black markers* indicate individual participant scores

The self-report indices of explicit bias also showed some of the highest levels of reliability: both measures of Warmth_*W*_ and Preference_*W*_ exceeded a standard of good reliability (0.60). For the SAE, ratings of empathic concern for the negative images (Concern_*NEG*_), and arousal for the positive (Arousal_*POS*_) and negative images (Arousal_*NEG*_) surpassed good levels of reliability, but ratings of empathic concern for positive images (Concern_*POS*_) reached a moderate level of reliability (> 0.40). For the DPT task, the SDI was the most reliable, reaching a standard of good reliability, whereas the focus and conflict indices both showed poor levels of reliability (< 0.40). As we also observed large variability in accuracy scores on the DPT task, we report the test–retest reliabilities for participants who performed this task with above 70% and 80% in File S1, which do not change our interpretations. Scatterplots for these tasks are shown in Fig. 3.

**Fig. 4:**
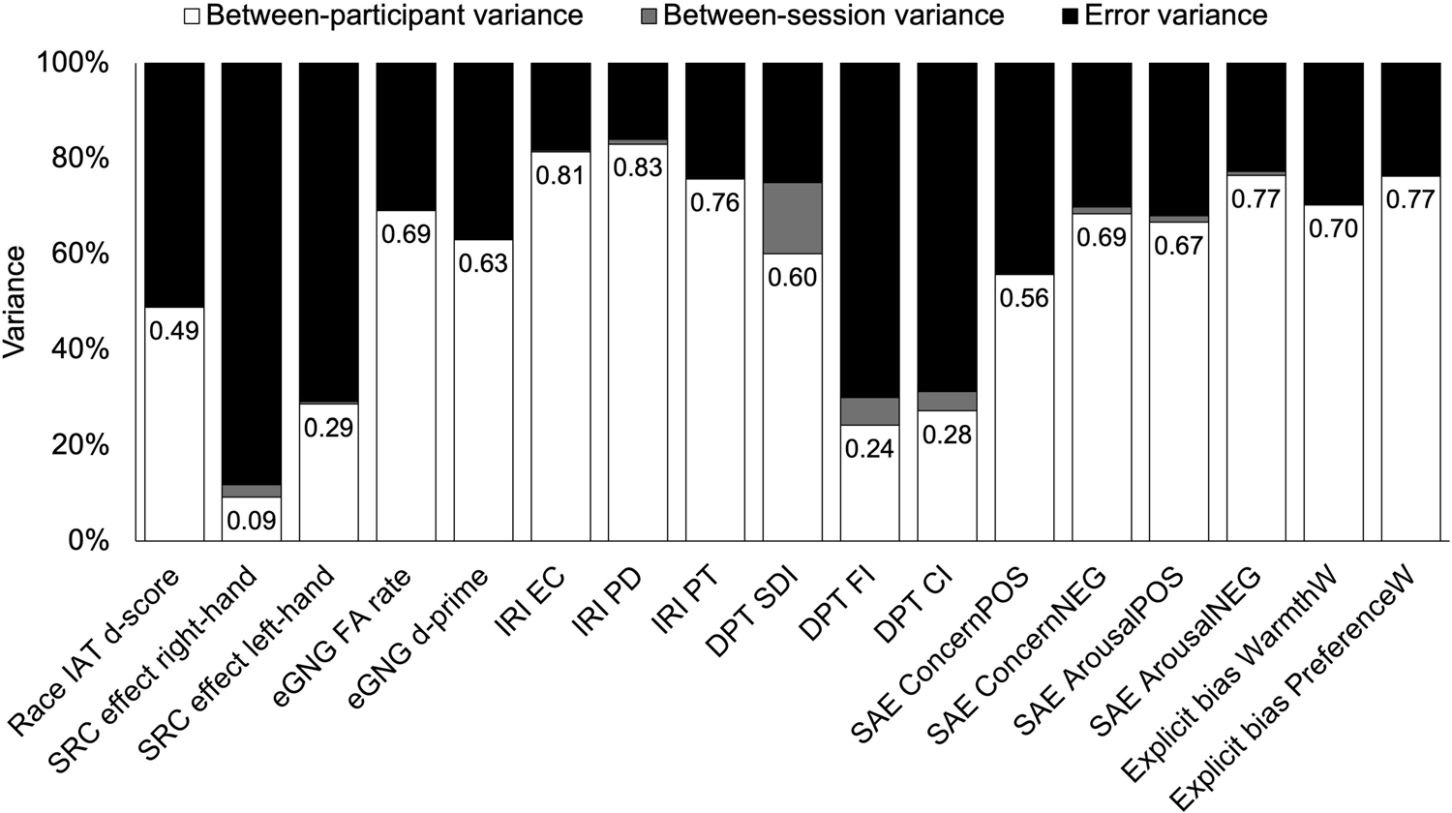
Variance components for task indices. *Note.* Bar sizes are normalised according to the total variance for the measure and subdivided into between-participant variance (*white*), between-session variance (*grey*) and error variance (*black*). The corresponding ICC is printed within each bar

In addition to the SEM, it is useful to look at the relative size of each variance component used to calculate the ICC. Figure 4 shows the proportion of variance accounted for by differences between participants, differences between sessions, and error for each task index. Measures with higher ICCs have a higher proportion of between-participant variance in contrast to the error variance; the opposite relationship is typically observed within measures with lower reliability (Clark et al., 2022; Hedge et al., 2018). This figure further illustrates that the poor reliability of some measures does not come from between-session (e.g., learning) effects.

## Discussion

Social cognitive abilities are crucial for healthy social functioning; to interact effectively with others, we must encode multiple social cues simultaneously (e.g., verbal and non-verbal expressions), infer their thoughts, emotions, and motivations, and adapt our behaviour in a context-appropriate manner. The importance of these socio-cognitive processes is evident in the interpersonal dysfunction that results from their impairment, recognised increasingly as a transdiagnostic criterion of most – if not all – psychiatric, neurological, and developmental disorders (see Cotter et al., 2018; Schilbach, 2016). Indeed, in the most recent version of the *Diagnostic and Statistical Manual for Mental Disorders* (DSM-5-TR; APA, 2022), the concept of social cognition has been introduced alongside memory and executive control as one of six core components that can be affected by neurocognitive disorder. Understanding the causes and consequences of disruptions to social cognition processes is therefore of crucial importance, but this requires accurate measurement tools capable of assessing individual differences.

Various experimental tasks have been developed to measure key socio-cognitive processes and researchers utilise these frequently to investigate individual differences (e.g., Bukowski & Samson, 2017; Happé et al., 2017; Shaw et al., 2020). However, whilst such tasks provide strong within-participant effects that make them excellent experimental paradigms, this can make them suboptimal for investigating individual differences that require high between-participant variability (Hedge et al., 2018). For the first time, we performed an evaluation of test–retest reliability for common experimental tasks that measure distinct components of social cognition: automatic imitation, perspective taking, empathy, emotion recognition, and implicit intergroup attitudes. Estimates of test–retest reliability varied considerably between the tasks and their various measurement indices: the Emotional Go/No-Go (eGNG) had good reliability (ICC = 0.63–0.69), the State Affective Empathy (SAE) task had moderate-to-good reliability (ICC = 0.56–0.77), the race-Implicit Association Test (r-IAT) had moderate reliability (ICC = 0.49), the Dot Perspective Taking (DPT) task had poor-to-good reliability (ICC = 0.24–0.60), and the Stimulus–Response Compatibility (SRC) task had poor reliability (ICC = 0.09–0.29). Lower estimates of test–retest reliability resulted from low between-subject variance and high error variance. Conversely, self-report indices had consistently better test–retest reliability: the subscales of the Interpersonal Reactivity Index (IRI) had good-to-excellent reliability (ICC = 0.76–0.83) and Explicit Bias (EB) had good reliability (ICC = 0.70 to 0.77) in line with previous research demonstrating that self-report measures tend to yield higher test–retest reliability than experimental measures (Hedge et al., 2018; Zeynep Enkavi et al., 2019). Researchers should provide an explicit rationale as to why they have selected a certain task and its indices for the assessment of individual differences, whilst also considering that acceptable reliability thresholds differ based on whether these measures are being used in experimental or clinical research. Although estimates of 0.60 are nominally viewed as good for the former, estimates of 0.80 are considered a clinically required standard (Cichetti & Parrow, 1981; Fleiss, 1981; Landis & Koch, 1977). As such, only indices with test–retest reliability estimates of > 0.60 should be considered appropriate to make stricter inferences about individual differences.

**Fig. 5:**
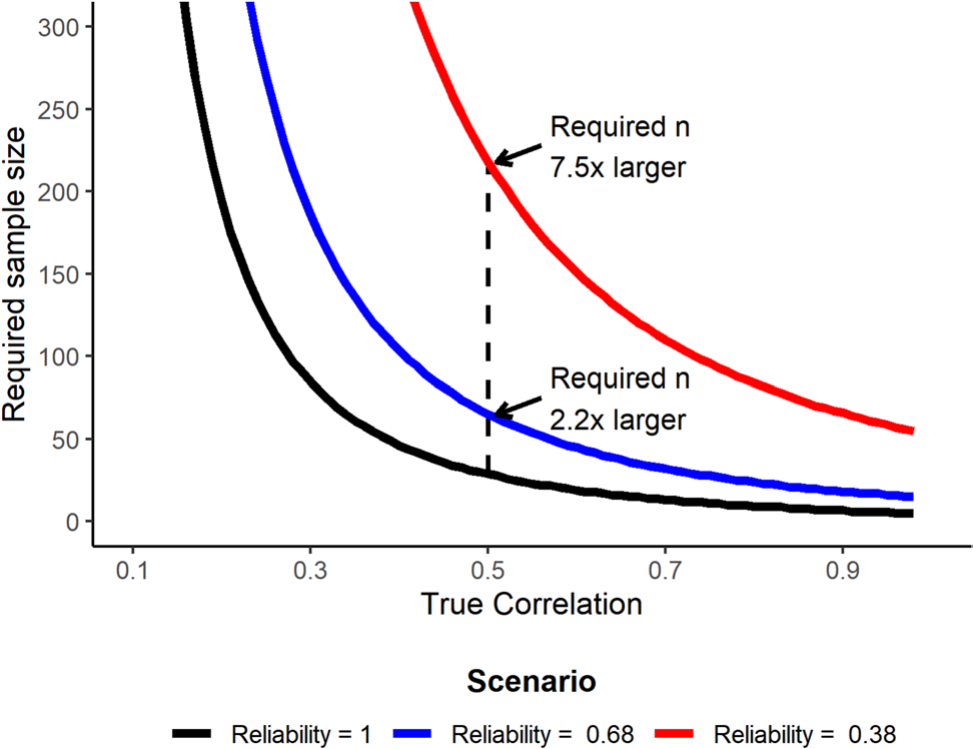
Sample sizes required for sufficient statistical power (80%) according to the true correlation and the reliability of measures. *Note.* The *black line* plots the sample size (*y*-axis) required for 80% power for a range of correlations (*x*-axis), assuming a two-tailed test and *α* = 0.05. The *black line* assumes that the two measures have perfect reliability, so the correlation observed in the data is equal to the true correlation. To construct the *blue line*, we calculated the equivalent attenuated observed correlation for two measures that have reliabilities of ICC = 0.77 and ICC = 0.60 and plot the sample sizes required for 80% power. The *red line* repeats this, assuming reliabilities of ICC = 0.29 and ICC = 0.49. The *black dashed line* highlights the differences in the sample sizes required for sufficient power for the same true correlation (*r* = 0.50, a strong effect) at different levels of reliability

Another reason why researchers should be interested in test–retest reliability is because lower reliability attenuates correlations between measurement indices, which increases the sample sizes required to achieve sufficient statistical power in correlational research (e.g.Hedge et al., 2018; Parsons et al., 2019; Spearman, 1910). Without consideration of reliability, then, researchers may not be able to discover the findings they set out to. It is useful to illustrate this in the context of the levels of reliability observed in the current study. Figure 5 plots the sample sizes required for 80% power to detect a statistically significant correlation of different strengths, assuming a two-tailed test and alpha of 0.05. The solid black line assumes perfect reliability in two measures, so the correlation that we expect to observe in the data is equal to the “true” correlation in the underlying dimensions. The blue and red lines start with the same true correlation and show the impact of two different levels of reliability on the observed correlation.^6^ For the blue line, we assume here that researchers aim to assess the relationship between the negative arousal index from the SAE task (ICC = 0.77 in our data) and the single dimension index from the DPT task (ICC = 0.60). While these levels of reliability typically fall in the range interpreted as “good” (Cicchetti & Sparrow, 1981; Fleiss, 1981; Landis & Koch, 1977), the sample sizes required for sufficient power are two to three times higher than if reliability was assumed to be perfect. The red line in Fig. 5 applies a similar logic, but taking the reliabilities observed for the SRC effect (left-hand, ICC = 0.29) and the r-IAT (ICC = 0.49). This leads to required sample sizes that are seven to eleven times higher. Clearly, the levels of reliability that we observe can impact how we design our studies and the size of correlations that we have sufficient power to detect. When conducting a power analysis to determine an appropriate sample size, researchers should consider whether they are identifying an effect that takes reliability into account (e.g. based on a previous study or meta-analysis), or an assumption about what the underlying true relationship is between the indices of interest.

Perhaps unsurprisingly, tasks that use difference scores as the main dependent variable showed the lowest levels of test–retest reliability. Specifically, the automatic imitation effect on the SRC task and the conflict and focus perspective indices on the DPT task were the lowest out of 17 indices estimated from the other experimental tasks and self-report measures. If two task indices are highly correlated and have similar variance, computing a difference score results in lower reliability than if these indices are used independently (Cronbach & Furby, 1970; Hedge et al., 2018). The main reason for this is that any subtraction that reduces between-participant variance can increase the proportion of measurement error relative to between-participant variance, which is unwanted in individual differences research and thus results in lower test–retest reliability estimates (Hedge et al., 2018). The poor test–retest reliabilities of these particular indices therefore render them unsuitable for testing individual differences, but does not preclude another reliable index from the same task to be used for this purpose. This is exemplified with the DPT task: whilst the test–retest reliability for the conflict and focus indices had poor reliability, the single-dimension index which does not utilise a difference score had good reliability.

Low test–retest reliability does not equate to a measure having low *validity* – indeed, these measures detect robust and replicable within-participant effects of their purported constructs, which is required in experimental research. The point is that robust experimental effects do not always translate well to studying individual differences. Research by Hedge et al. (2018) on the *reliability paradox* explains that this is likely because experimental tasks have been developed and selected because they produce robust within-participant effects, which often means low between-participant variance. When tasks have low between-participant variance, it is difficult to reliably distinguish between individuals, and test–retest reliability tends to be low. Measures with poor reliability are therefore unsuitable for tests of individual differences because the ability to detect relationships with other constructs will be compromised by the inability to distinguish effectively between individuals on that dimension (Spearman, 1910). To capture reliable individual differences in social cognition, we need experimental measurement tools that capture between-participant variability in each constituent process. This means that the assessment of test–retest reliability should be routine practice in task development (Hedge et al., 2020). Moreover, since reliability is a product of the population sample and not the measure itself, it should ideally be measured and reported in each study investigating individual differences.

Some of our tasks deserve additional consideration because they have recently been the subject of intense scrutiny within the social cognition literature, and our findings contribute to these debates further. Several studies have reported the test–retest reliability of the (race) IAT and our point estimate (ICC = 0.49, *r* = 0.51) lies within the meta-analytic confidence interval bounds reported by Lai and Wilson (2021; *r* = 0.49, 95% CI = 0.38–0.59). Whilst it is important to note that such estimates vary based on the duration between task administration, the sample sizes obtained, and the way in which the IAT is scored (see Lane et al., 2007; Kvam et al., 2024), it appears overall, then, that this task has lower-than-desired test–retest reliability making it suboptimal as an individual difference measure of implicit intergroup bias (see also Connor & Evers, 2020). This is particularly important to emphasise given that the race-IAT has made societal impact: people completing this task via the Project Implicit website receive personalised feedback on their IAT score, yet recent research has shown that these scores have extremely large confidence intervals that means such individual-level inferences are inappropriate (Cummins & Hussey, 2023; Klein, 2020). Furthermore, we found higher test–retest estimates for our indices of Explicit Racial Bias in line with previous research indicating that implicit measures show lower stability over time than conceptually corresponding explicit measures, despite comparable estimates of internal consistency (Gawronski et al., 2017). If researchers wish to use the IAT, then they should be aware that our data suggests that only half of the variance from this task represents individual differences, and this will likely underestimate the true relationship between intergroup bias and other measures of socio-cognitive processes due to attenuation.

The SRC task, which is proposed to measure automatic imitation, showed the lowest reliability out of the five experimental tasks that we employed. This was despite using two variations of task stimuli – one that is used most frequently to avoid simple spatial-compatibility effects (Brass, 2001; see Cracco et al., 2018a, 2018b; Heyes, 2011) and another that avoids both spatial and orthogonal-compatibility effects (Czekóová et al., 2021; Shaw et al., 2017). Task difficulty did not appear to explain this, with reliability remaining low when we adjusted the inclusion criterion from > 50% accuracy to > 70 and > 80% accuracy (see File S1). Recently, this measure has come under further scrutiny, with studies also documenting low split-half reliability (Pennington et al., 2023) and issues of construct validity, suggesting that rather than measuring mechanisms specialised for social information processing the SRC may measure more general cognitive processes that support response inhibition and interference resolution (Czekóová et al., 2021; Darda et al., 2020). Interestingly, the negative automatic imitation score observed in session 2 for the right-hand stimulus suggests that participants were influenced more by some (non-social) spatial aspect of this task rather than the actions they were observing.

## Limitations and future directions

The findings of the current study should be interpreted in line with sample generalisability, several methodological design decisions, and other factors that may have influenced our estimates of test–retest reliability.

Our sample were mainly young females, predominantly white, and from the United Kingdom. As such, it is unknown whether similar test–retest reliability estimates would be identified across varying demographic factors of gender, age, ethnicity, and culture. Future research should endeavour to evaluate task reliability *and* validity to ensure psychological science can improve the generalisability of its measures (see, for example, Oshiro et al., 2024).

When deciding on the stimuli to use for the DPT task, we employed photographs of human actors in real-life settings who were facing the camera and turning their heads to look at either the left or right wall. However, such stimuli differ from the original Samson et al. (2010) study for two reasons – (1) they used computerised avatars; and (2) both the avatar’s head and body were oriented laterally so that they were facing the right or left wall. The decision to use human actors was based on the work of Langton (2018) who argues that this is a more valid test of visual perspective taking, and the decision to only orient the actor’s head was to reduce the potential confound that (non-social) attentional cuing can exert on perspective taking (see Conway et al., 2017). Nevertheless, such design decisions may influence task difficulty and test–retest estimates. Indeed, the error rate for the DPT task in the current study exceeded 10% meaning that we could not calculate inverse efficiency scores that are frequently used as an index of perspective-taking ability. Psychological scientists should endeavour to continuously evaluate reliability in the task development phase based on these different task design decisions.

Of the 17 indices selected here, six showed potential practice effects. Participants were quicker to respond on the DPT task and showed less egocentric interference on this task in session 2 compared to session 1. They also reported lower personal distress on the IRI, and lower negative and positive arousal, and negative concern on the SAE in session 2. These indices resulted in good-to-excellent test–retest reliability, which raises the question as to whether practice effects are responsible for this. Nevertheless, other indices that did not show practice effects, such as for eGNG task, IRI, and EB, also showed good-to-excellent reliability, suggesting that practice effects did not unduly affect test–retest reliability in the current study.

Finally, test–retest estimates will differ based on whether the measured constructs represent trait or state characteristics; since traits are enduring characteristics that are stable over time, they tend to yield higher estimates of test–retest reliability compared to states that are temporary and/or flexible (Brysbaert, 2024). Informing the rationale for the current study, much of the literature points towards a trait-based explanation of social cognition, with researchers proposing taxonomies or structures of interdependent processes, akin to personality and intelligence (see Happé et al., 2017; Nosek et al., 2011; Shaw et al., 2020; Schimmack, 2021a, 2021b for critical discussions). In this way, research proposes that components of social cognition can establish the parameters that quantify (potentially heritable) normative and aberrant behaviours (e.g., Gur & Gur, 2015; see also DSM-5-TR; APA, 2022). However, it is important to consider that some research suggests specific sociocognitive processes, such as automatic imitation and perspective taking, can be trained (Cook et al., 2010; Oliveros et al., 2024; Wiggett et al., 2011) and are influenced by experience and mood (e.g., Catmur et al., 2009; Heyes et al., 2005). This implies that states, or a combination of trait and state processes, underpin social cognition, which has implications for the test–retest reliability of experimental measures. Overall, though, this appears to be an explicitly unanswered question in the field, marking a significant direction for future research.

## Conclusion

Experimental social cognition tasks are employed routinely to assess individual differences, but their suitability for this is rarely evaluated. In the current study, we performed the first large-scale assessment of test–retest reliability for an extensive battery of tasks designed to assess the socio-cognitive processes of automatic imitation, emotion recognition, empathy, perspective taking, and intergroup bias. Our findings provide further empirical support for the *reliability paradox* (Hedge et al., 2018), highlighting how some indices that show robust within-participant effects in experimental designs are sub-optimal for assessing individual differences. Researchers interested in answering fundamental questions about intra-individual variability in social cognition must report the test–retest reliability of their measures; only with accurate measurement tools can we make credible claims about human behaviour.

## Supplementary Information

Below is the link to the electronic supplementary material.

Supplementary file1 (DOCX 9717 KB)

## Data Availability

All experimental materials, data, and analysis scripts are publicly available via the Open Science Framework: https://osf.io/q569f/

## Footnotes

1 Earlier work using the Dot Perspective-Taking Task suggested that the consistency effect shown repeatedly on this task provides evidence of implicit mentalising, or Theory of Mind. However, such interpretation has been challenged in more recent studies (see Conway et al., 2017; Holland et al., 2021). As such, in the current study we stipulate that this task measures Level-1 perspective taking, as proposed in the original study by Samson et al. (2010), and do not refer to implicit mentalising.

2 According to a Google Scholar search conducted on 06/08/2024, these tasks have yielded the following citation counts: IAT, *n* = 17,590 times (Greenwald et al., 1998); Stimulus–Response Compatibility task, *n* = 934 (Brass, 2001); Emotional Go/No-Go, *n* = 444 (Tottenham et al., 2011); Dot Perspective Taking task, *n* = 859 times (Samson et al., 2010); and State Affective Empathy task (also known as Multifaceted Empathy task), *n* = 1225 (Dziobek et al., 2008). Although these citation counts do not inform us about how many times these tasks have been used, they speak to their popularity in the field of experimental social cognition.

3 We reflect on this design choice and its potential influence on test–retest reliability estimates in the Discussion.

4 Due to a computer error, participants only saw 13 negative images; however, this error was present for both sessions and therefore has no impact on test–retest reliability estimates.

5 For the DPT task performance check, ‘Self’ and ‘Other’ perspective trials were collapsed to report the main effect of Consistency (Consistent vs. Inconsistent) for match trials. For the SAE performance check, the measures of empathic concern and arousal were aggregated and compared between positive and negative valence images.

6 Observed *r* = True r * sqrt(reliability x * reliability y).

## Code availability

All analysis scripts are publicly available at: https://osf.io/q569f/
