# Supplementary material for: Are we capturing individual differences? Evaluating the test–retest reliability of experimental tasks used to measure social cognitive abilities
Source: Behav Res Methods. 2025 Jan 31;57(2):82. doi: 10.3758/s13428-025-02606-5 (PMC11785611; doi:10.3758/s13428-025-02606-5)
Supplement: Supplementary file 1 — Supplementary file1 (DOCX 9717 KB) [file 13428_2025_2606_MOESM1_ESM.docx]

**Supplementary Information**

**Are we capturing individual differences? Evaluating the test-rest reliability of common experimental tasks used to measure social cognitive abilities.**

Charlotte R. Pennington^1^, Kayley Birch-Hurst^1,2^, Matthew Ploszajski^2,3^, Kait Clark^2^, Craig Hedge^1^, & Daniel J. Shaw^1^

^1^Department of Psychology, School of Health & Life Sciences, Aston University, Birmingham, UK.

^2^School of Social Sciences, University of the West of England, Bristol, UK.

^3^Department of Computer Science, College of Science and Engineering, Swansea University, Wales, UK.

*****Correspondence regarding this article should be addressed to: Dr Charlotte R. Pennington, School of Psychology, College of Health & Life Sciences, Aston University, Birmingham, B4 7ET. E-mail: [c.pennington@aston.ac.uk](mailto:c.pennington@aston.ac.uk)

**Contents**

1. **Data Exclusions**
2. **Performance and Replication Checks**
3. **Exploratory Reliability Estimates**
4. **Data Exclusions**

One-hundred and sixty-two participants were recruited to take part in a study investigating relationships between different measures of social cognition. Twelve participants did not return for session two (attrition rate = 7.41%). Individual task/questionnaire data was excluded from analyses in two iterative steps: first, if either timepoint was missing for the task/questionnaire, and second, if participants scored below 50% accuracy on the SRC or DPT (tasks which typically require above chance performance). Participant exclusions for each of these two steps are detailed as follows:

| **Step 1: Missing data** |  |
| --- | --- |
| *Individual task data exclusion across sessions* |  |
| Session 1, Race IAT = 1 |  |
| Session 1, SRC right-hand = 0 |  |
| Session 1, SRC left-hand = 0 |  |
| Session 1, eGNG = 0 |  |
| Session 1, IRI = 2 |  |
| Session 1, DPT = 6 |  |
| Session 1, SAE = 3 |  |
| Session 1, Explicit bias = 4 |  |
|  | **Did participants match across S1-S2?** |
| Session 2, Race IAT = 2 | No, all additional participants |
| Session 2, SRC right-hand = 2 | No, all additional participants |
| Session 2, SRC left-hand = 2 | No, all additional participants |
| Session 2, eGNG = 2 | No, all additional participants |
| Session 2, IRI = 4 | No, all additional participants |
| Session 2, DPT = 4 | 2 across T1-T2, 2 additional participants |
| Session 2, SAE = 4 | 2 across T1-T2, 2 additional participants |
| Session 2, Explicit bias = 2 | No, all additional participants |
|  |  |
| **Step 2: Accuracy data** |  |
| Individual task data exclusion across sessions, <50% accuracy |  |
| Session 1, SRC right-hand = 31 |  |
| Session 1, SRC left-hand = 4 |  |
| Session 1, DPT = 7 |  |
|  | **Did participants match across T1-T2?** |
| Session 2, SRC right-hand = 15 | No, all additional participants |
| Session 2, SRC left-hand = 3 | No, all additional participants |
| Session 2, DPT = 6 | No, all additional participants |

1. **Performance and Replication Checks**

For the four experimental tasks, all expected effects were observed and were consistent with previous studies. Specifically, participants exhibited a pro-White/anti-Black bias (*M* = 0.36, *SD* = 0.30) on the Race IAT, with the average *d*-score aggregated across sessions differing significantly from zero (*t*(146) = 14.71, *p* < .001, *d_z_* = 1.21). This is in line with Greenwald et al. (1998; Experiment 3) who also found participants displayed an implicit attitudinal preference for White over Black people on this task and is in line with the effect sizes reported by Greenwald et al. (2003). On the SRC, participants were quicker to respond to Compatible (*M =* 557.89, *SD* = 62.88) relative to Incompatible trials for the (orthogonally confounded) left-hand stimulus (*M* = 584.84, *SD* = 66.65; *t*(140) = 9.71, *p* < .001, *d_z_* = 0.82), in line with the meta-analysis by Cracco et al. (2018). However, the difference was non-significant between Incompatible (*M* = 545.45, *SD* = 65.76) and Compatible trials for the (non-confounded) right-hand stimulus (*M* = 540.71, *SD* = 66.46; *t*(101) = 1.58, *p* = 0.117, *d_z_* = 0.16). This latter result is somewhat expected given the absence of orthogonal confounds that has been shown to reduce – or even partially reverse – the automatic imitation effect (Czekóová et al., 2021). On the eGNG, participants had significantly higher false alarm rates on emotional (*M* = 0.32, *SD* = 0.14) relative to neutral No-Go trials (*M* = 0.14, *SD* = 0.10), *t*(149) = 21.55, *p* < .001, *d_z_* = 1.76), which is in line with the findings reported by Tottenham et al. (2011). On the DTP, participants responded quicker on Consistent^[[1]](#footnote-1)^ (*M* = 713.46, *SD* = 130.87) relative to Inconsistent trials (*M* = 796.93, *SD* = 144.00; *t*(128) = 15.30, *p* < .001, *d_z_* = 1.35), in line with effects reported by Samson et al. (2010) and Simpson and Todd (2017). On the DTP, participants also responded quicker to Self (*M* = 745.64, *SD* = 132.98) compared to Other trials (*M* = 764.75, *SD* = 139.91; *t*(128) = 4.23, *p* < .001, *d_z_* = 0.37). On the SAE, self-reported affective empathy was greater for negative (*M* = 4.54, *SD* = 1.28) relative to positive valence images (*M* = 2.17, *SD* = 0.91; *t*(144) = 21.61, *p* < .001, *d_z_* = 1.79).

1. **Exploratory Reliability Estimates**

As we observed large variability in performance on the Stimulus Response Compatibility (SRC) task and Dot Perspective (DPT) task, we also explored reliability estimates using stricter inclusion criteria for performance accuracy. Table S1 provides the test-retest reliabilities for participants who performed with above 70% and 80% accuracy for the main outcome variables on each of these tasks.

**Table S1**

*Test-Retest Reliability Estimates for Performance above 70% and 80% Accuracy for measures on the Stimulus Response Compatibility (SRC) Task and Dot Perspective-Taking (DPT) Task.*

| **Task** | **Measure** | **Accuracy >70%** | | **Accuracy >80%** | |
| --- | --- | --- | --- | --- | --- |
|  |  | ICC [68% CI] | Rho [95% CI] | ICC [68% CI] | Rho [95% CI] |
| SRC right-hand | SRC effect (ms) | 0.22 [0.02,0.40] | 0.13  [-0.08, 0.33] | 0.21  [0.01, 0.40] | 0.10  [-0.11, 0.31] |
| SRC left-hand | -- | 0.28 [0.12,0.43] | 0.25*  [0.08, 0.40] | 0.31 [0.14,0.45] | 0.27*  [0.10, 0.42] |
| DPT | SDI (ms) | 0.65 [0.20,0.83] | 0.73**  [0.61, 0.82] | 0.71  [0.29, 0.88] | 0.81**  [0.63, 0.91] |
|  | Focus index (ms) | 0.30  [0.08, 0.49] | 0.34*  [0.13, 0.52] | 0.22  [-0.13, 0.53] | 0.28  [-0.10, 0.60] |
|  | Conflict index (ms) | 0.35  [0.15, 0.52] | 0.36**  [0.15, 0.54] | 0.52  [0.18, 0.74] | 0.48*  [0.12, 0.72] |

*Note.* **p* < 0.01, ***p* < 0.001.

In comparison to our main analyses which included participants with above 50% accuracy, including participants with above 70% or 80% accuracy improved test-retest reliability estimates for the SRC task with the right-hand (orthogonally confounded) stimulus slightly but, overall, these estimates are still poor. Reliability estimates for the SRC task with the left-hand stimulus remained similar. Therefore, increasing the accuracy threshold for this task does not appear to have a substantial impact on reliability estimates and comes at the cost of excluding between 6.86% and 12.75% of additional data, respectively. Scatterplots for the SRC task are shown in Figure S1.

For the DPT task including participants with above 70% or 80% accuracy further improved the test-retest reliability estimate of the single dimension index (SDI) measure (ICC = 0.65, 0.71, respectively) but this remained within the threshold of good reliability. Increasing accuracy to >70% also improved reliability for the focus index measure (ICC = 0.30) but this was still within the poor threshold, and at >80% these estimates remained similar to those reported in our main analyses (ICC = 0.22). Finally, increasing accuracy to >70% had little effect on the conflict index measure with this still showing poor reliability (ICC = 0.35), but reliability increased for this measure at >80% accuracy from poor to moderate (ICC = 0.52). However, to achieve >70% accuracy on the DPT task we had to exclude 34.88% of data, and to achieve >80% accuracy we had to exclude 77.52% - the majority of our data! As such, the slight increases in test-retest reliability estimates for some of our task measures comes at the cost of excluding a lot of the data.

**Figure S1**

*Scatterplots for Performance above 70% and 80% Accuracy for measures from the Stimulus Response Compatibility (SRC) Task.*


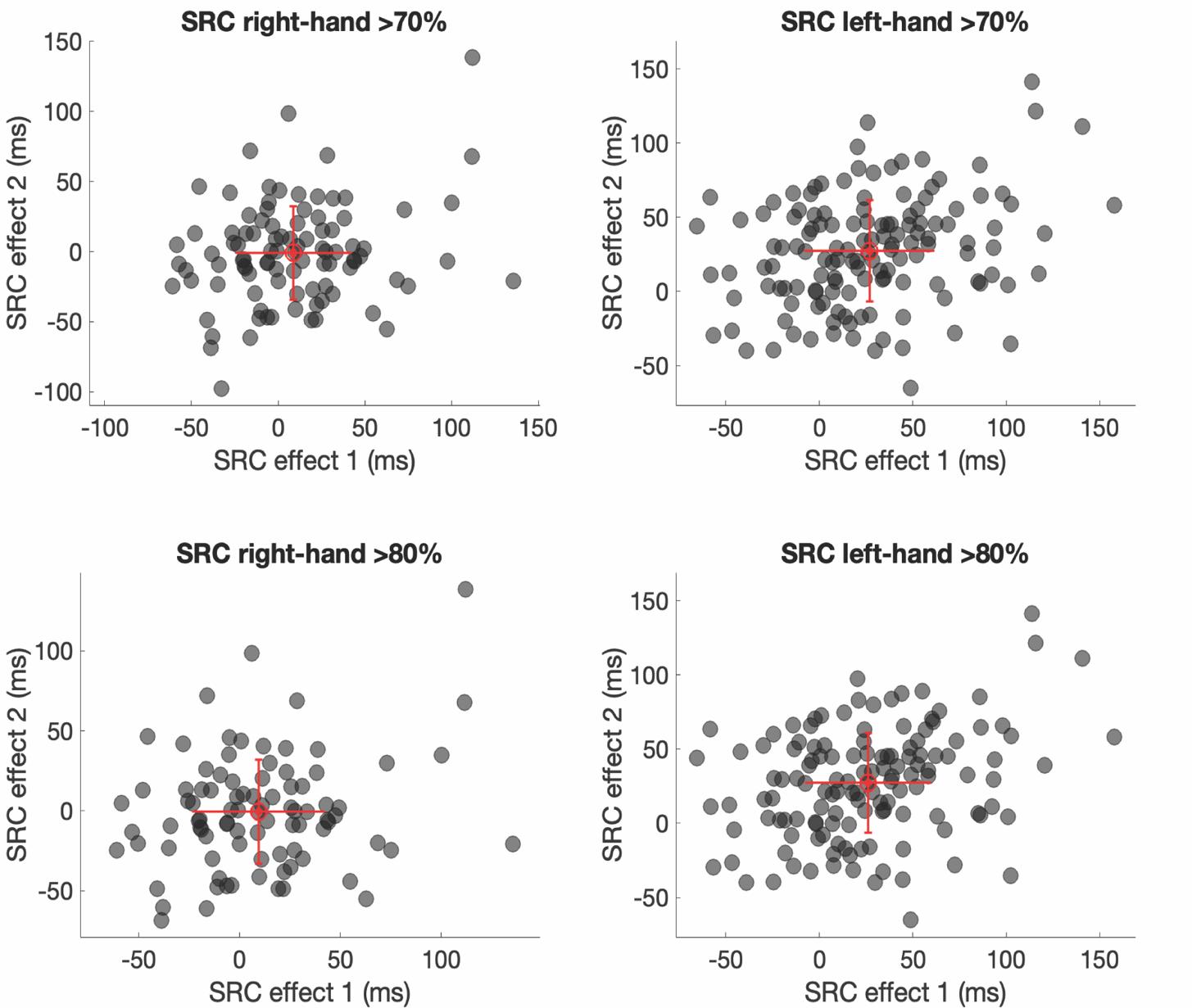


*Note.* SRC right-hand >70% = Stimulus Response Compatibility task with right-hand stimulus and participants who performed above 70% accuracy (*n* = 95); SRC left-hand >70% = Stimulus Compatibility task with left-hand stimulus and participants who performed above 70% accuracy (*n* = 136); SRC right-hand >80% = Stimulus Response Compatibility task with right-hand stimulus and participants who performed above 80% accuracy (*n* = 89); SRC left-hand >80% = Stimulus Compatibility task with left-hand stimulus and participants who performed above 80% accuracy (*n* = 133). Red markers indicate mean group performance from sessions 1 and 2. Error bars show ± standard error of measurement (SEM). Black markers indicate individual participant scores.

**Figure S2**

**
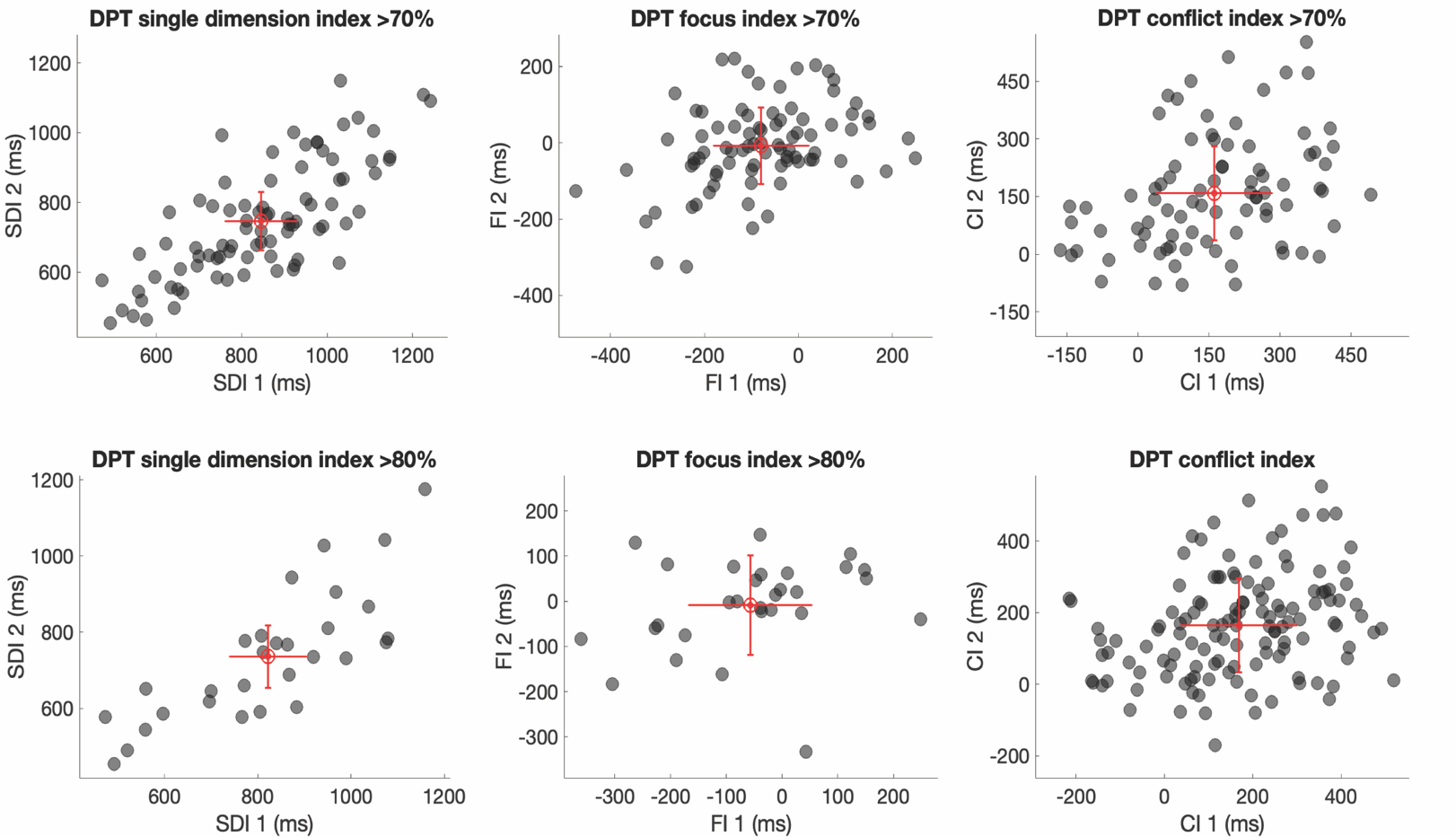
***Scatterplots for Performance above 70% and 80% Accuracy for measures from the Dot Perspective-Taking (DPT) Task.*

*Note.* DPT = Dot Perspective-Taking task, SDI = Single-dimension index, FI = Focus index, CI = Conflict index; >70% = participants who performed above 70% accuracy (*n* = 84); >80% = participants who performed above 80% accuracy (*n* = 29). Red markers indicate mean group performance from sessions 1 and 2. Error bars show ± standard error of measurement (SEM). Black markers indicate individual participant scores.

1. For the DPT performance check, ‘Self’ and ‘Other’ perspective trials were aggregated to report the main effect of Consistency (Consistent vs. Inconsistent) for match trials. For the SAE performance check, the measures of empathic concern and arousal were aggregated and compared between positive and negative valence images. [↑](#footnote-ref-1)
